# Multiple Myeloma Breast Involvement: A Case Report

**DOI:** 10.1155/2019/2079439

**Published:** 2019-10-09

**Authors:** Heba O. E. Ali, Zafar Nasir, Ahmed M. S. M. Marzouk

**Affiliations:** ^1^Consultant Breast Radiology, Altnagelvin Area Hospital, WHSC Trust, Glenshane Rd, Londonderry BT47 6SB, UK; ^2^Consultant Radiology, Altnagelvin Area Hospital, WHSC Trust, Glenshane Rd, Londonderry BT47 6SB, UK; ^3^Consultant General Surgery, Altnagelvin Area Hospital, WHSC Trust, Glenshane Rd, Londonderry BT47 6SB, UK; ^4^Consultant General Surgery, Faculty of Medicine, Cairo University, Al-Saray Street, El Manial, Cairo 11956, Egypt

## Abstract

Multiple Myeloma involving the breast is very rare and the diagnosis is challenging because the clinical and radiological features of breast multiple myeloma are indistinguishable to other forms of breast disease whether primary or metastatic. In this article the authors report a case presented with breast masses, which were found to be extra osseous Multiple Myeloma. The patient was managed for multiple spinal lesions that were primarily thought to be metastasis from primary breast cancer.

## 1. Introduction

Multiple myeloma (MM) is a hematological malignancy characterized by the clonal proliferation of plasma cells in the bone marrow with increased formation of monoclonal immunoglobulins [1]. The average patient age at diagnosis is sixth decade of life, and the disease is very rarely seen in patients aged <40 years. MM can affect extraosseous sites as solitary lesions (extramedullary plasmacytoma) or as a presentation of MM relapse in less than 5% of cases. Moreover, MM in the breast has been rarely documented [2–4]; the first case of MM in the breast was reported in 1925 [4]. Since then, only 20 other patients with breast involvement have been documented in the literature till date [5]. Here we report an unusual presentation of MM, focusing on the imaging findings.

## 2. Case Report

**Figure 1:**
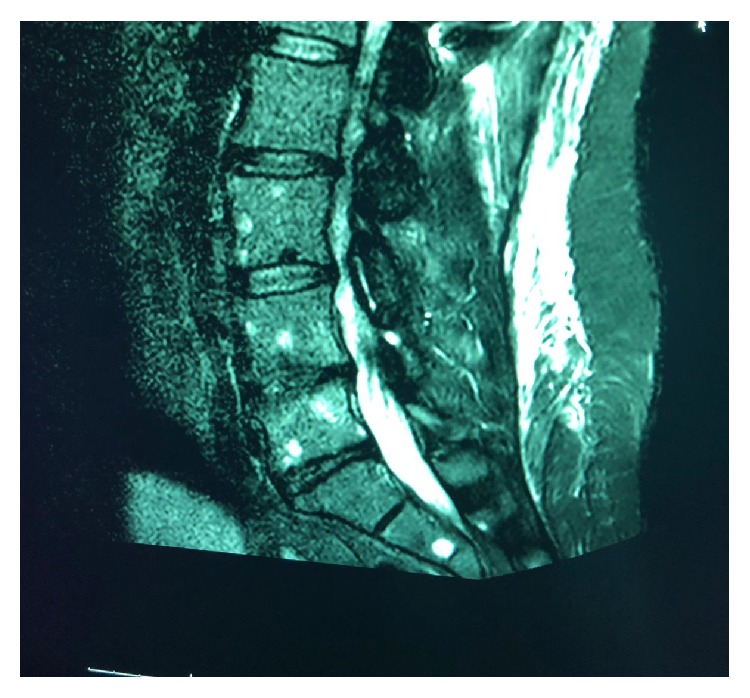
MRI shows multiple, variable-sized, well-defined round lesions.

**Figure 2:**
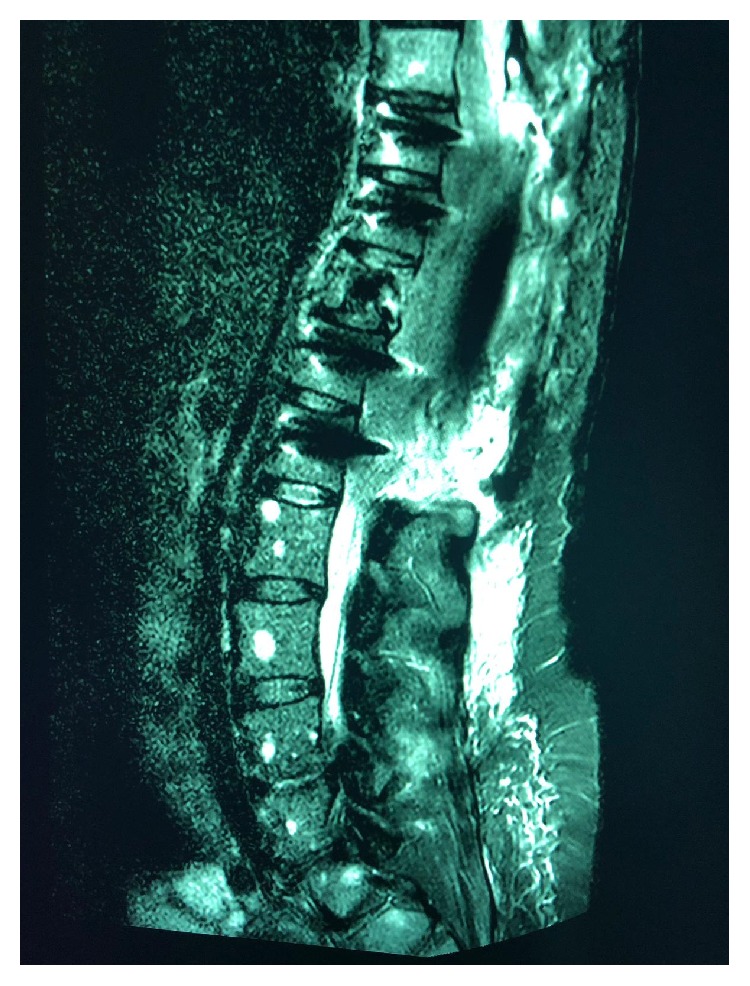
Post-spinal decompression and spinal fixation MRI.

A 40-year-old woman complaining of severe back pain for several months, which then progressed to bilateral lower limb weakness, visited our hospital. Her medical history was unremarkable. Initial magnetic resonance imaging (MRI) of the spine revealed multiple, variable-sized, well-defined, round lesions with soft tissue masses compressing the spinal cord (Figure 1). Spinal fixation was performed (Figure 2). The initial diagnosis was considered to be metastasis of unknown origin. Clinical examination revealed a lump in the left breast, which was believed to be the primary breast neoplasm and was further investigated; meanwhile, bone biopsy was arranged.

**Figure 3:**
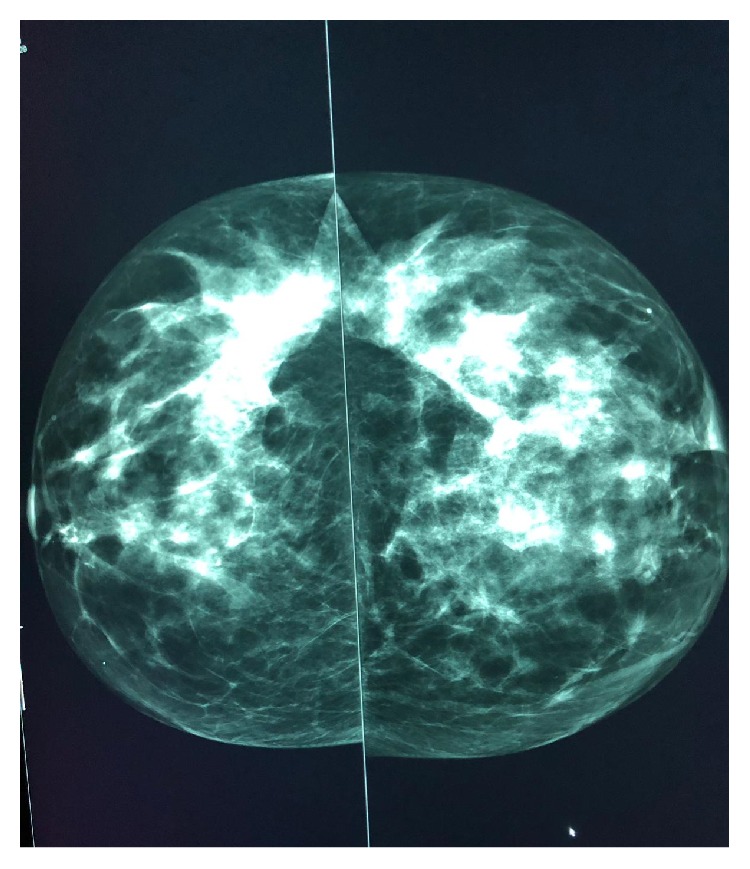
Mammogram CC view showing dense breasts (inconclusive).

**Figure 4:**
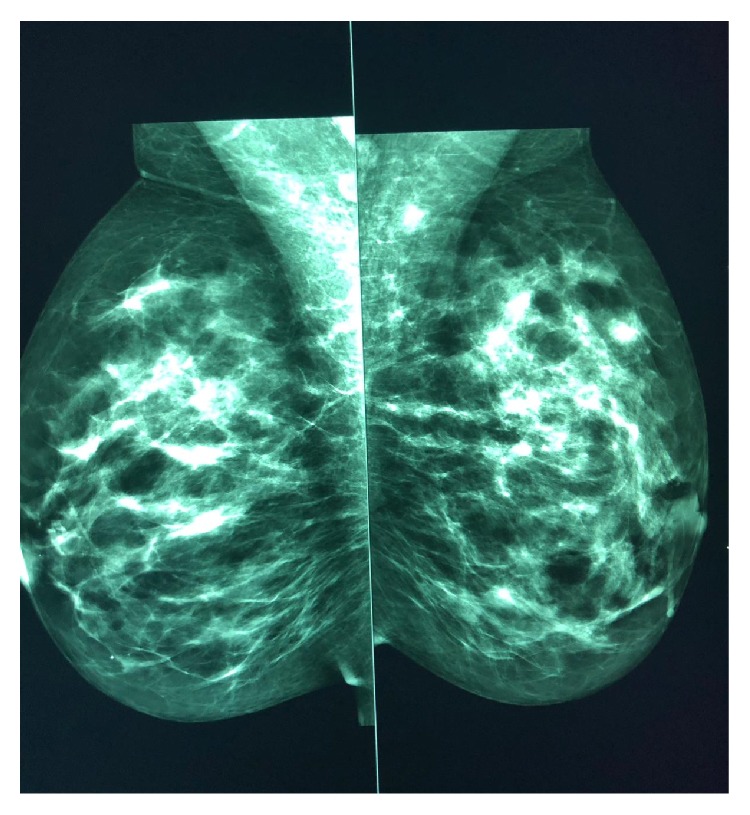
Mammogram MLO view showing dense breasts (inconclusive).

**Figure 5:**
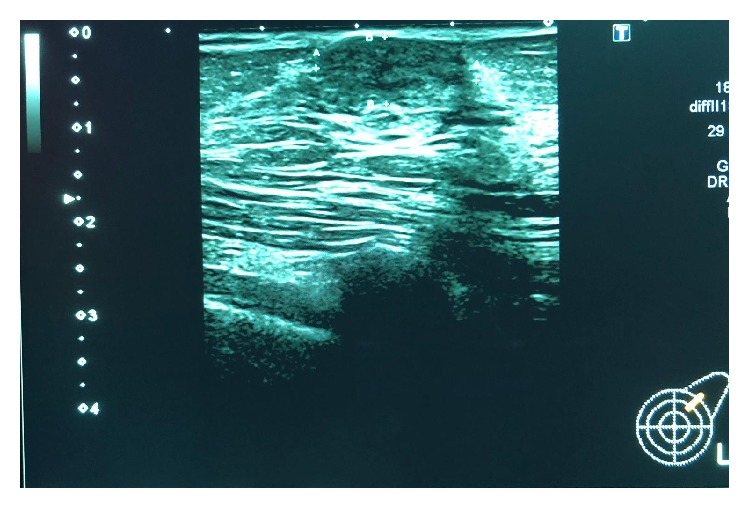
Ultrasound of the left breast showing a well-defined hypoechoic solid mass lesion, measuring 16 mm × 7 mm.

**Figure 6:**
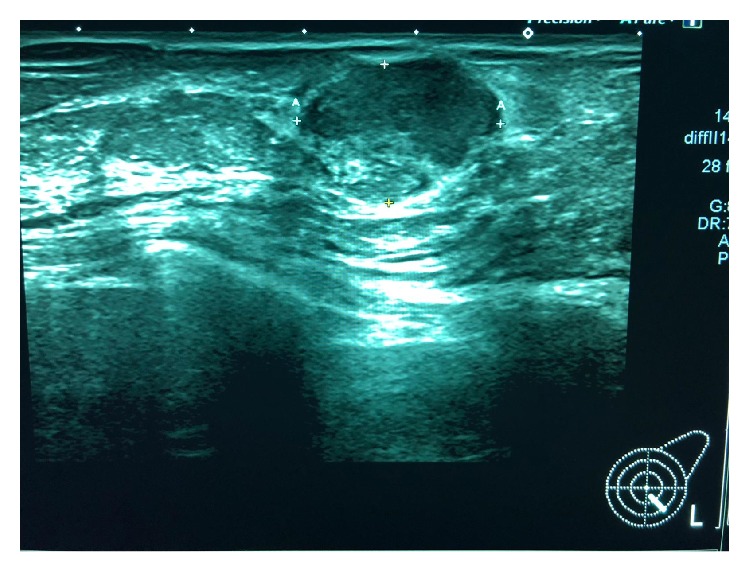
Ultrasound of the left breast showing a second well-defined hypoechoic solid mass, measuring 18 mm × 13 mm.

**Figure 7:**
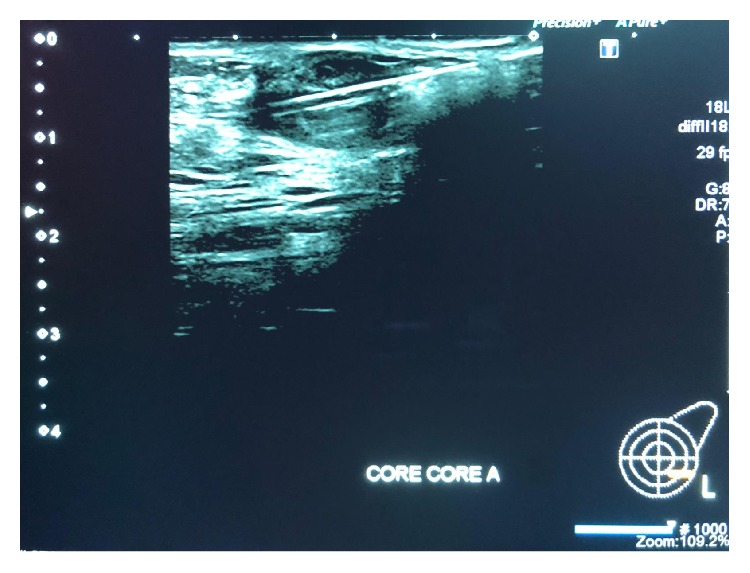
Ultrasound-guided core biopsy image of the first lesion (14-gauge needle).

**Figure 8:**
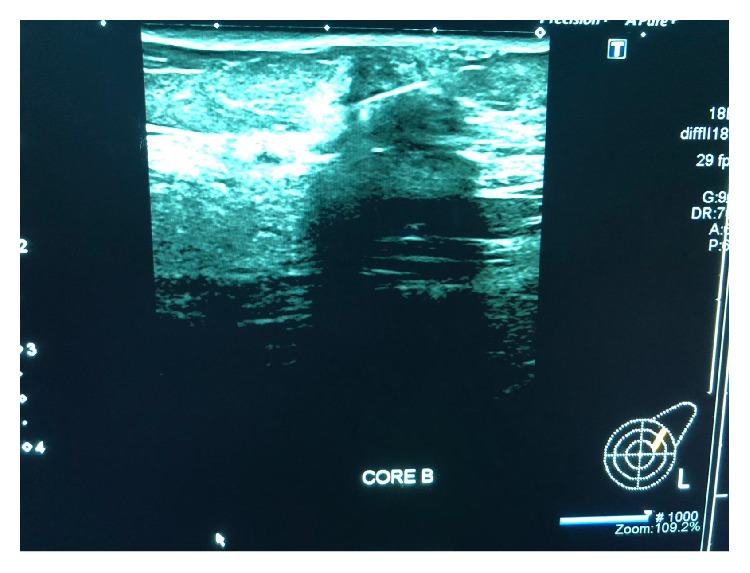
Ultrasound-guided core biopsy of the second lesion (14-gauge needle).

Mammography was performed, which revealed dense breasts (Figures 3 and 4). Ultrasonography (US) of both breasts revealed a well-defined hypoechoic solid lesion in the left breast 4 o'clock in location at the site of the clinically palpable lump, measuring 18 mm × 13 mm; US revealed a second similar lesion at 1 o'clock in location, measuring 16 mm × 7 mm (Figures 5 and 6); these lesions were both classified as U3. Subsequently, US-guided core biopsies of both lesions were performed (Figures 7 and 8).

Pathological examination revealed large sheets of atypical plasma cells, which displayed rounded nuclei with coarse chromatin and conspicuous nucleoli; abundant eosinophilic cytoplasm was present in some cells. Mitotic figures and apoptotic cells were readily identified. Immunohistochemistry revealed that the atypical cells were positive for CD138 and CD56 and focally positive for CD79a; the cells were lambda restricted. The appearance was entirely consistent with a plasma cell neoplasm involving the breast. The features were similar in both breast lesions.

**Figure 9:**
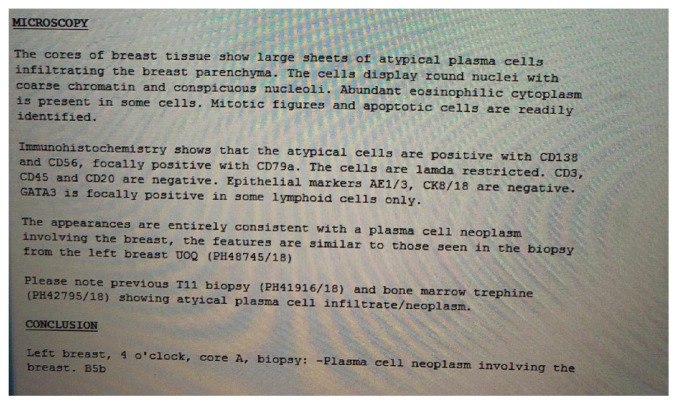
Pathology report.

T11 biopsy and bone marrow trephine revealed atypical plasma cell infiltrate/neoplasm (Figure 9).

## 3. Discussion

MM, a disease of plasma cells, affects individuals in their middle age with an incidence of 3–4 cases/100,000 individuals in the United States population. A majority of the patients with plasma cell neoplasia present with generalized disease at diagnosis; a minority of patients present with a single extramedullary mass of monoclonal plasma cells (plasmacytoma) either in bone (97%) or soft tissues (3%) which may present as solitary lesion or as a relapse of MM which is explained by clonal evolution due to variety of theories [6]. A breast mass is a very rare presentation in MM, and most plasmacytomas in the breast have been identified in women with a mean age of presentation of 53 years [6–10].

Breast MM can be single or multiple. Unilateral and bilateral presentations have been reported, with lesion sizes ranging between 1 cm and 7.5 cm; further, axillary lymph node involvement has been reported [8]. These tumors may present as solitary plasmacytic tumors without the evidence of concurrent MM or can precede, occur at the same time, or present after the diagnosis of MM [9]. An average time of 1.5–2.5 years is needed by 30%–50% of extramedullary plasmacytoma cases to progress to MM [11].

In this report, the patient showed unusual presentation because she was young; MM is typically a disease of older adults with the median age at diagnosis being 66 years. Only 10%, 2%, and 0.3% of patients are younger than the ages of 50, 40, and 30 years, respectively [12, 13]. Clinically, most patients with MM in the breast will present with a palpable mass; however, skin thickening and inflammation can occur and be confused with breast abscess or inflammatory carcinoma [14]. The differential diagnosis of such a mass that presents within the clinical context of plasma cell neoplasms includes primary epithelial neoplasm of the breast, plasma cell mastitis, nonHodgkin's lymphoma with plasmacytic features, epithelioid malignant melanoma, and pseudolymphoma [15]. The features of MM in the breast (Table 1) are indistinguishable from those of the other forms of breast diseases, whether primary or metastatic; therefore, same imaging protocol that is applied for any suspicious breast mass is used for plasmacytic tumors. In a mammogram, MM can present as single or multiple well-defined, ill-defined, or speculate mass lesions or even with microcalcifications. which represent nonspecific findings [7]. In our case, the mammogram was inconclusive owing to the patient's age.

US findings typically include well-defined hypoechoic or hyperechoic solid mass lesions [7]; in our case, the results of US resembled a fibroadenoma. In MRI findings, MM appears hypointense on T1WIs and hyperintense on T2WIs and shows early ring enhancement with washout in postcontrast images [7]. Breast plasmacytomas demonstrate low-grade uptake of 18-fluorodeoxyglucose, but PET-CT can assess the extent of disease [14].

Because mammography, US, and MRI findings may not be diagnostic, the differential diagnosis for primary carcinoma, other lymphoproliferative diseases, and even benign masses depends on histopathological evaluation [4]. Breast MM/plasmacytoma treatment depends on the systemic extent of the disease. Although chemotherapy and radiotherapy are the most frequent treatment options, surgical resection and lymph node dissection can also be considered [8, 13–17].

## 4. Conclusion

In conclusion, there are no definite radiological features for MM in the breast. When multiple breast masses are detected, secondary involvement of a hematological disorder, metastatic malignancies, and benign diseases, such as fibroadenoma, should be considered in the differential diagnosis. In this case, mammography and US findings were inconclusive, and the diagnosis was made using tissue biopsy. Proper diagnosis changed the disease management in this case, and systemic chemotherapy was initiated.

## Figures and Tables

**Table 1:** Radiological features of plasmacytoma breast lesions.

Possible radiological features of plasmacytoma breast lesions (Nonspecific)
*Mammogram*
(i) Hyperdense, round or oval, masses with well- or ill-defined margins.
(ii) Diffuse infiltration.
(iii) Microcalcifications (rare).
*Ultrasound*
(i) Echo-poor or hypoechoic well-defined masses with hypervascularity.
(ii) Mixed hypo- to hyperechoic lesions with indistinct margins.
(iii) Posterior acoustic features:
(1) Enhanced or no acoustic transmission.
(2) Posterior acoustic shadowing.
*Magnetic resonance*
(i) Hypointense on T1WIs.
(ii) Hyperintense on T2WIs.
(iii) Early ring enhancement with washout in postcontrast images.
